# Supplementary material for: Observation of full-parameter Jones matrix in bilayer metasurface
Source: Nat Commun. 2022 Dec 7;13:7550. doi: 10.1038/s41467-022-35313-2 (PMC9729203; doi:10.1038/s41467-022-35313-2)
Supplement: Supplementary file 1 — supplementary information [file 41467_2022_35313_MOESM1_ESM.pdf]

Supplementary Information for

Observation of Full-Parameter Jones Matrix in Bilayer Metasurface

Yanjun Bao[1,*], Fan Nan[1], Jiahao Yan[1], Xianguang Yang[1], Cheng-Wei Qiu[2,*] and Baojun Li[1,*]

[1]Institute of Nanophotonics, Jinan University, Guangzhou 511443, China

[2]Department of Electrical and Computer Engineering, National University of Singapore, Singapore 117583, Singapore

*Corresponding Authors: Y. Bao (yanjunbao@jnu.edu.cn), C.-W. Qiu (chengwei.qiu@nus.edu.sg), B. Li (baojunli@jnu.edu.cn)

Table of Contents

S1. Derivation of Eq. (1)

S2. The detail of the gradient descent optimization

S3. Precondition of Eq. 2 and boundary constraint in gradient descent optimization

S4. Design of the target equivalent Jones matrix and optimized Jones matrix components of single layers

S5. Metasurface unit design

S6. FDTD simulation of the realistic structures with different optical setups

S7. Effects of detour phase in multi-element unit design

S8. Alignment sensitivity of the two layer metasurfaces

S9. Optical setup for measurement

S10. Calculation of optical responses with rotation between the two layer metasurfaces

S11. Optical performance of the two layer metasurfaces designed with different gap distance

S1. Derivation of Eq. (1)

We consider an optical system composed of two layer metasurfaces that are separated by a distance z (Figure S1). The distributions of the Jones matrix values are denoted as $J^1 = \begin{bmatrix} J_{11}^1(x_1,y_1) & J_{12}^1(x_1,y_1) \\ J_{21}^1(x_1,y_1) & J_{22}^1(x_1,y_1) \end{bmatrix}$ and $J^2 = \begin{bmatrix} J_{11}^2(x_2,y_2) & J_{12}^2(x_2,y_2) \\ J_{21}^2(x_2,y_2) & J_{22}^2(x_2,y_2) \end{bmatrix}$ for metasurfaces 1 and 2, respectively, where (x_1, y_1) and (x_2, y_2) are the xy coordinates. As the single metasurface layer has mirror symmetry with respect to the transverse plane, the off-diagonal elements of the Jones matrix should be identical, i.e, $J_{12}^1(x_1,y_1) = J_{21}^1(x_1,y_1)$ and $J_{12}^2(x_2,y_2) = J_{21}^2(x_2,y_2)$.

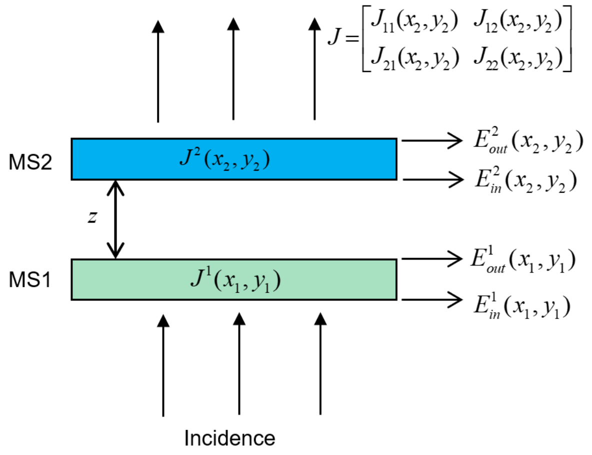

Figure S1. Schematic view of a two layer metasurface system separated by a vertical distance z. The Jones matrixes of the two single layers are denoted as $J^1(x_1,y_1)$ and $J^2(x_2,y_2)$, respectively. The light is incident from the bottom of metasurface 1, with the electric fields at the incident and output planes of metasurface 1 denoted by $E_{in}^1(x_1,y_1)$ and $E_{out}^1(x_1,y_1)$, the electric fields at the incident and output planes of metasurface 2 denoted by $E_{in}^2(x_2,y_2)$ and $E_{out}^2(x_2,y_2)$, respectively. J represents the equivalent Jones matrix of the whole structure. MS: metasurface.

We then derive the equivalent Jones matrix expression $J(x_2, y_2)$ of the whole two-layer optical system. Considering the light is firstly incident on the metasurface 1 with polarization along x direction $E_{in}^1 = [1\ \ 0]^T$, the electric field distributions passing through metasurface 1 is

$$E_{out}^1(x_1, y_1) = \begin{bmatrix} J_{11}^1(x_1, y_1) & J_{12}^1(x_1, y_1) \\ J_{21}^1(x_1, y_1) & J_{22}^1(x_1, y_1) \end{bmatrix} \begin{bmatrix} 1 \\ 0 \end{bmatrix} = \begin{bmatrix} J_{11}^1(x_1, y_1) \\ J_{21}^1(x_1, y_1) \end{bmatrix} \tag{S1.1}$$

After propagating a distance z, the electric field distributions $E_{in}^2(x_2, y_2)$ at the bottom surface of metasurface 2 is

$$E_{in}^2(x_2, y_2) = \begin{bmatrix} \iint\limits_{x_1, y_1} J_{11}^1(x_1, y_1) \cdot f(x_2 - x_1, y_2 - y_1, z) dx_1 dy_1 \\ \iint\limits_{x_1, y_1} J_{21}^1(x_1, y_1) \cdot f(x_2 - x_1, y_2 - y_1, z) dx_1 dy_1 \end{bmatrix} \tag{S1.2}$$

where we have used the Rayleigh–Sommerfeld diffraction formula to calculate the light propagation, $f(x_2 - x_1, y_2 - y_1, z) = \frac{1}{2\pi} \frac{\exp(ikr)}{r} \frac{z}{r} \left(\frac{1}{r} - i\frac{2\pi}{\lambda} \right)$ is the Rayleigh–Sommerfeld impulse response, $r = \sqrt{(x_1 - x_2)^2 + (y_1 - y_2)^2 + z^2}$, i is the imaginary unit, λ is the wavelength and z is the distance between the two layers.

The electric field passing through metasurface 2 at the output plane is

$$\begin{aligned} E_{out}^2(x_2, y_2) &= J^2(x_2, y_2) \cdot E_{in}^2(x_2, y_2) \\ &= \begin{bmatrix} \sum\limits_{q=1,2} J_{1q}^2(x_2, y_2) \iint\limits_{x_1, y_1} J_{q1}^1(x_1, y_1) \cdot f(x_2 - x_1, y_2 - y_1, z) dx_1 dy_1 \\ \sum\limits_{q=1,2} J_{2q}^2(x_2, y_2) \iint\limits_{x_1, y_1} J_{q1}^1(x_1, y_1) \cdot f(x_2 - x_1, y_2 - y_1, z) dx_1 dy_1 \end{bmatrix} \end{aligned} \tag{S1.3}$$

Similarly, we can obtain the electric fields at the output plane of metasurface 2 with y-polarized incidence $E_{in}^1 = [0\ 1]^T$ as

$$E_{out}^{2}(x_2,y_2)=\begin{bmatrix}\sum_{q=1,2} J_{1q}^{2}(x_2,y_2)\iint_{x_1,y_1} J_{q2}^{1}(x_1,y_1)\cdot f(x_2-x_1,y_2-y_1,z)dx_1dy_1\\ \sum_{q=1,2} J_{2q}^{2}(x_2,y_2)\iint_{x_1,y_1} J_{q2}^{1}(x_1,y_1)\cdot f(x_2-x_1,y_2-y_1,z)dx_1dy_1\end{bmatrix} \quad \text{(S1.4)}$$

Based on Eqs. S1.3 and 1.4, we can obtain the equivalent Jones matrix of the two layer system with its *mn* (*m*, *n*=1, 2) component as

$$J_{mn}(x_2,y_2)=\sum_{q=1,2} J_{mq}^{2}(x_2,y_2)\iint_{x_1,y_1} J_{qn}^{1}(x_1,y_1)\cdot f(x_2-x_1,y_2-y_1,z)dx_1dy_1 \quad \text{(S1.5)}$$

This is the derivation of Eq. 1 in the main text.

Alternatively, one can use the angular spectrum method to calculate the field propagation between the two layers. For this case, Equation S1.5 becomes

$$J_{mn}(x_2,y_2)=\sum_{q=1,2} J_{mq}^{2}(x_2,y_2)\iint_{u,v} A_{qn}^{1}(u,v,z)\cdot \exp(i2\pi(ux_2+vy_2))dudv \quad \text{(S1.6)}$$

where $A_{qn}^{1}(u,v,z)=A_{qn}^{1}(u,v,0)G(u,v,z)$, $G(u,v,z)=\exp(i2\pi z(\lambda^{-2}-u^2-v^2)^{1/2})$ and $A_{qn}^{1}(u,v,0)=\iint_{x_1,y_1} J_{qn}^{1}(x_1,y_1)\cdot \exp(-i2\pi(ux_1+vy_1))dx_1dy_1$.

The two methods are equivalent. However, for numerical calculation, errors are introduced due to the discretization of the functions. The two methods are applicable to in different scenes. For example, for the same sampling intervals, the Rayleigh–Sommerfeld diffraction is suitable for long distance propagation[1] while the angular spectrum method is suitable for near field regions[2]. For our structures, the metasurface has a length size of hundred micrometers and sampled interval of 0.8 μm. If we want to calculate the light propagation between the two layers (with a gap distance of 150 micrometers), the angular spectrum method is more suitable. For the calculation of the

holography which is designed at a distance of few thousand of micrometers above the metasurface 2, the Rayleigh–Sommerfeld diffraction method is more suitable. As the expression of the Rayleigh–Sommerfeld diffraction is more concise, we use it in the main text and the following derivations.

S2. The detail of the gradient descent optimization

In this section, we provide the detail of the gradient descent optimization method to obtain the Jones matrix distributions of the two layer metasurfaces $J^1(x_1, y_1)$ and $J^2(x_2, y_2)$ to design a target equivalent Jones matrix distribution $J^t(x_2, y_2)$. For numerical calculations, all the planes are sampled to $N \times N$ equidistant grids with sampling intervals of $\Delta x = \Delta y = P$. We define a loss of

$$L_{mase} = \frac{1}{4N^2} \sum_{m=1}^{2} \sum_{n=1}^{2} \sum_{i=1}^{N} \sum_{j=1}^{N} \left| J_{mn}(x_{2i}, y_{2j}) - J^t_{mn}(x_{2i}, y_{2j}) \right|^2 \tag{S2.1}$$

which is the mean of the absolute squared error between the target J^t_{mn} and the calculated Jones matrixes J_{mn} of all the four components. Here, J_{mn} is a function of the Jones matrix components of the two single layers (see Eq. S1.5 or Eq. S1.6). The core step of the gradient descent optimization method is to calculate the gradient of the defined loss with respect to the input variables, i.e., the Jones matrix components of the two single layers. Note that all the Jones matrix components are complex values and therefore have two independent variables. Take the *11*th component of the Jones matrix of the first layer metasurface for example, it is written as $J^1_{11}(x_{1p}, y_{1q}) = u^1_{11}(x_{1p}, y_{1q}) + iv^1_{11}(x_{1p}, y_{1q})$ and the gradient of L_{mase} with respect to

$u_{11}^1(x_{1i}, y_{1j})$ and $v_{11}^1(x_{1i}, y_{1j})$ can be calculated as

$$\frac{\partial L_{mase}}{\partial u_{11}^1(x_{1p}, y_{1q})} = 2\,\mathrm{Re}\left(\frac{\partial L_{mase}}{\partial J_{11}^1(x_{1p}, y_{1q})}\frac{\partial J_{11}^1(x_{1p}, y_{1q})}{\partial u_{11}^1(x_{1p}, y_{1q})}\right) = 2\,\mathrm{Re}\left(\frac{\partial L_{mase}}{\partial J_{11}^1(x_{1p}, y_{1q})}\right)$$

$$= 2\,\mathrm{Re}\left(\sum_{m=1}^{2}\sum_{n=1}^{2}\sum_{i=1}^{N}\sum_{j=1}^{N}\frac{1}{4N^2}\left(J_{mn}(x_{2i}, y_{2j}) - J_{mn}^t(x_{2i}, y_{2j})\right)^* \frac{\partial J_{mn}(x_{2i}, y_{2j})}{\partial J_{11}^1(x_{1p}, y_{1q})}\right)$$

$$= 2\,\mathrm{Re}\left(\sum_{m=1}^{2}\sum_{i=1}^{N}\sum_{j=1}^{N}\frac{1}{4N^2}\left(J_{m1}(x_{2i}, y_{2j}) - J_{m1}^t(x_{2i}, y_{2j})\right)^* \frac{\partial J_{m1}(x_{2i}, y_{2j})}{\partial J_{11}^1(x_{1p}, y_{1q})}\right)$$

$$= \frac{1}{2N^2}\mathrm{Re}\left(\sum_{m=1}^{2}\sum_{i=1}^{N}\sum_{j=1}^{N}\left(J_{m1}(x_{2i}, y_{2j}) - J_{m1}^t(x_{2i}, y_{2j})\right)^* J_{m1}^2(x_{2i}, y_{2j}) f(x_{2i} - x_{1p}, y_{2j} - y_{1q}, z)\Delta x \Delta y\right)$$

$$\frac{\partial L_{mase}}{\partial v_{11}^1(x_{1p}, y_{1q})}$$

$$= \frac{1}{2N^2}\mathrm{Re}\left(i\sum_{m=1}^{2}\sum_{i=1}^{N}\sum_{j=1}^{N}\left(J_{m1}(x_{2i}, y_{2j}) - J_{m1}^t(x_{2i}, y_{2j})\right)^* J_{m1}^2(x_{2i}, y_{2j}) f(x_{2i} - x_{1p}, y_{2j} - y_{1q}, z)\Delta x \Delta y\right)$$

where *p*, *q* are the coordinate indexes of first layer and *i, j* are the coordinate indexes of the second layer.

As $J_{12}^1(x_1, y_1) = J_{21}^1(x_1, y_1)$, the gradient of *F* with respect to J_{12}^1 (or J_{21}^1) should be the sum of the gradients with respect to J_{12}^1 and J_{21}^1, that is

$$\frac{\partial L_{mase}}{\partial u_{12}^1(x_{1p}, y_{1q})}$$

$$= 2\,\mathrm{Re}\left(\frac{\partial F}{\partial J_{12}^1(x_{1p}, y_{1q})}\frac{\partial J_{12}^1(x_{1p}, y_{1q})}{\partial u_{12}^1(x_{1p}, y_{1q})}\right) + 2\,\mathrm{Re}\left(\frac{\partial F}{\partial J_{21}^1(x_{1p}, y_{1q})}\frac{\partial J_{21}^1(x_{1p}, y_{1q})}{\partial u_{21}^1(x_{1p}, y_{1q})}\right)$$

$$= \frac{1}{2N^2}\mathrm{Re}\left(\sum_{m=1}^{2}\sum_{i=1}^{N}\sum_{j=1}^{N}\left(J_{m2}(x_{2i}, y_{2j}) - J_{m2}^t(x_{2i}, y_{2j})\right)^* J_{m1}^2(x_{2i}, y_{2j}) f(x_{2i} - x_{1p}, y_{2j} - y_{1q}, z)\Delta x \Delta y\right)$$

$$+ \frac{1}{2N^2}\mathrm{Re}\left(\sum_{m=1}^{2}\sum_{i=1}^{N}\sum_{j=1}^{N}\left(J_{m1}(x_{2i}, y_{2j}) - J_{m1}^t(x_{2i}, y_{2j})\right)^* J_{m2}^2(x_{2i}, y_{2j}) f(x_{2i} - x_{1p}, y_{2j} - y_{1q}, z)\Delta x \Delta y\right)$$

$$\frac{\partial L_{mase}}{\partial v_{12}^1(x_{1p}, y_{1q})} =$$

$$=\frac{1}{2N^2}\mathrm{Re}\left(i\sum_{m=1}^{2}\sum_{i=1}^{N}\sum_{j=1}^{N}\left(J_{m2}(x_{2i},y_{2j})-J_{m2}^{t}(x_{2i},y_{2j})\right)^{*}J_{m1}^{2}(x_{2i},y_{2j})f(x_{2i}-x_{1p},y_{2j}-y_{1q},z)\Delta x\Delta y\right)$$

$$+\frac{1}{2N^2}\mathrm{Re}\left(i\sum_{m=1}^{2}\sum_{i=1}^{N}\sum_{j=1}^{N}\left(J_{m1}(x_{2i},y_{2j})-J_{m1}^{t}(x_{2i},y_{2j})\right)^{*}J_{m2}^{2}(x_{2i},y_{2j})f(x_{2i}-x_{1p},y_{2j}-y_{1q},z)\Delta x\Delta y\right)$$

Similarly, we have

$$\frac{\partial L_{mase}}{\partial u_{22}^{1}(x_{1p},y_{1q})}$$
$$=\frac{1}{2N^2}\mathrm{Re}\left(\sum_{m=1}^{2}\sum_{i=1}^{N}\sum_{j=1}^{N}\left(J_{m2}(x_{2i},y_{2j})-J_{m2}^{t}(x_{2i},y_{2j})\right)^{*}J_{m2}^{2}(x_{2i},y_{2j})f(x_{2i}-x_{1p},y_{2j}-y_{1q},z)\Delta x\Delta y\right)$$

$$\frac{\partial L_{mase}}{\partial v_{22}^{1}(x_{1p},y_{1q})}$$
$$=\frac{1}{2N^2}\mathrm{Re}\left(i\sum_{m=1}^{2}\sum_{i=1}^{N}\sum_{j=1}^{N}\left(J_{m2}(x_{2i},y_{2j})-J_{m2}^{t}(x_{2i},y_{2j})\right)^{*}J_{m2}^{2}(x_{2i},y_{2j})f(x_{2i}-x_{1p},y_{2j}-y_{1q},z)\Delta x\Delta y\right)$$

The gradients of L_{mase} with respect to the components of the Jones matrix of the second layer $J^{2}(x_2,y_2)$ can be calculated directly as

$$\frac{\partial L_{mase}}{\partial u_{11}^{2}(x_{2p},y_{2q})}$$
$$=\frac{1}{2N^2}\mathrm{Re}\left(\sum_{m=1}^{2}\sum_{i=1}^{N}\sum_{j=1}^{N}\left(J_{1m}(x_{2p},y_{2q})-J_{1m}^{t}(x_{2p},y_{2q})\right)^{*}J_{1m}^{1}(x_{1i},y_{1j})f(x_{2p}-x_{1i},y_{2q}-y_{1j},z)\Delta x\Delta y\right)$$

$$\frac{\partial L_{mase}}{\partial v_{11}^{2}(x_{2p},y_{2q})}$$
$$=\frac{1}{2N^2}\mathrm{Re}\left(i\sum_{m=1}^{2}\sum_{i=1}^{N}\sum_{j=1}^{N}\left(J_{1m}(x_{2p},y_{2q})-J_{1m}^{t}(x_{2p},y_{2q})\right)^{*}J_{1m}^{1}(x_{1i},y_{1j})f(x_{2p}-x_{1i},y_{2q}-y_{1j},z)\Delta x\Delta y\right)$$

$$\frac{\partial L_{mase}}{\partial u_{12}^{2}(x_{2p},y_{2q})}$$

$$=\frac{1}{2N^2}\mathrm{Re}\left(\sum_{m=1}^{2}\sum_{i=1}^{N}\sum_{j=1}^{N}\left(J_{1m}(x_{2p},y_{2q})-J_{1m}^{t}(x_{2p},y_{2q})\right)^{*}J_{2m}^{1}(x_{1i},y_{1j})f(x_{2p}-x_{1i},y_{2q}-y_{1j},z)\Delta x\Delta y\right)$$

$$+\frac{1}{2N^2}\mathrm{Re}\left(\sum_{m=1}^{2}\sum_{i=1}^{N}\sum_{j=1}^{N}\left(J_{2m}(x_{2p},y_{2q})-J_{2m}^{t}(x_{2p},y_{2q})\right)^{*}J_{1m}^{1}(x_{1i},y_{1j})f(x_{2p}-x_{1i},y_{2q}-y_{1j},z)\Delta x\Delta y\right)$$

$$\frac{\partial L_{mase}}{\partial v_{12}^{2}(x_{2p},y_{2q})}=$$

$$=\frac{1}{2N^2}\mathrm{Re}\left(i\sum_{m=1}^{2}\sum_{i=1}^{N}\sum_{j=1}^{N}\left(J_{1m}(x_{2p},y_{2q})-J_{1m}^{t}(x_{2p},y_{2q})\right)^{*}J_{2m}^{1}(x_{1i},y_{1j})f(x_{2p}-x_{1i},y_{2q}-y_{1j},z)\Delta x\Delta y\right)$$

$$+\frac{1}{2N^2}\mathrm{Re}\left(i\sum_{m=1}^{2}\sum_{i=1}^{N}\sum_{j=1}^{N}\left(J_{2m}(x_{2i},y_{2j})-J_{2m}^{t}(x_{2i},y_{2j})\right)^{*}J_{1m}^{2}(x_{2i},y_{2j})f(x_{2i}-x_{1p},y_{2j}-y_{1q},z)\Delta x\Delta y\right)$$

$$\frac{\partial L_{mase}}{\partial u_{22}^{2}(x_{2p},y_{2q})}$$

$$=\frac{1}{2N^2}\mathrm{Re}\left(\sum_{m=1}^{2}\sum_{i=1}^{N}\sum_{j=1}^{N}\left(J_{2m}(x_{2p},y_{2q})-J_{2m}^{t}(x_{2p},y_{2q})\right)^{*}J_{2m}^{1}(x_{1i},y_{1j})f(x_{2p}-x_{1i},y_{2q}-y_{1j},z)\Delta x\Delta y\right)$$

$$\frac{\partial L_{mase}}{\partial v_{22}^{2}(x_{2p},y_{2q})}$$

$$=\frac{1}{2N^2}\mathrm{Re}\left(i\sum_{m=1}^{2}\sum_{i=1}^{N}\sum_{j=1}^{N}\left(J_{2m}(x_{2p},y_{2q})-J_{2m}^{t}(x_{2p},y_{2q})\right)^{*}J_{2m}^{1}(x_{1i},y_{1j})f(x_{2p}-x_{1i},y_{2q}-y_{1j},z)\Delta x\Delta y\right)$$

Besides the mean of the absolute squared error L_{mase}, we also add a boundary constraint loss L_{bnd} into the total loss, i.e., $L=L_{mase}+L_{bnd}$. The definition and the gradient of the boundary loss are provided in the next supplementary section. Then we use an optimization algorithm L-BFGS method to minimize the total loss. For our case, this algorithm starts with an initial estimate of the input variables, i.e., all the components Jones matrixes of the two single layer metasurfaces, and proceeds iteratively to refine the input variables with a sequence of better estimates. The derivatives of the total loss are used as a key driver of the algorithm to identify the direction of steepest descent.

S3. Precondition of Eq. 2 and boundary constraint in gradient descent optimization

The Jones matrixes of the two single layer metasurfaces are all symmetric. We consider a 2×2 complex symmetric matrix *J*, and figure out the preconditions that it can be decomposed into the summation of two symmetric unitary matrixes as the following form:

$$J=\begin{bmatrix} a\ b \\ b\ c \end{bmatrix}=R(-\theta_1)\begin{bmatrix} e^{i\varphi_1} & 0 \\ 0 & e^{i\varphi_2} \end{bmatrix}R(\theta_1)+R(-\theta_2)\begin{bmatrix} e^{i\varphi_3} & 0 \\ 0 & e^{i\varphi_4} \end{bmatrix}R(\theta_2) \qquad \text{S3.1}$$

where $R(\theta)=\begin{bmatrix} \cos\theta & -\sin\theta \\ \sin\theta & \cos\theta \end{bmatrix}$ is the rotation matrix. It should be noted that the main results have been presented in the supplementary materials of reference[3], but missing some specific details, which will be discussed here.

Theorem 1. A 2×2 symmetric unitary matrix J can be factorized in the form $J=W\begin{bmatrix} e^{i\varphi_1} & 0 \\ 0 & e^{i\varphi_2} \end{bmatrix}W^{-1}$, where *W* is unitary and can be chosen as real orthogonal matrix, i.e, $W=\begin{bmatrix} \cos\theta & -\sin\theta \\ \sin\theta & \cos\theta \end{bmatrix}$.

Proof: It is known that a unitary matrix is unitarily diagonalizable and its eigenvalues are unimodular, therefore $J=W\Lambda W^{-1}$, $\Lambda=\begin{bmatrix} e^{i\varphi_1} & 0 \\ 0 & e^{i\varphi_2} \end{bmatrix}$ and $WW^{\dagger}=I$. Here $W^{\dagger}$ is the conjugate transpose of *W*.

Since J is also symmetric, there exist real symmetric matrices *A* and *B* such that

$J = A + iB$. Then $J^{\dagger}J = \left(A - iB\right)\left(A + iB\right) = A^2 + B^2 + i(AB - BA) = I$, which imply that A and B commute $AB = BA$. As real symmetric matrix can have real eigenvectors, a pair of real commuting symmetric matrices can be simultaneously diagonalized by the same set of real eigenvectors, which are also the eigenvectors of J. Therefore, W can be real matrix. Also, a general expression of a 2 × 2 unitary matrix can be written in the form:

$$W = e^{i\alpha/2}\begin{bmatrix} e^{i\alpha_1}\cos\theta & e^{i\alpha_2}\sin\theta \\ -e^{-i\alpha_2}\sin\theta & e^{-i\alpha_1}\cos\theta \end{bmatrix} \qquad \text{S3.2}$$

When it is real, it becomes $W = \begin{bmatrix} \cos\theta & -\sin\theta \\ \sin\theta & \cos\theta \end{bmatrix}$.

Theorem 2. A 2×2 symmetric matrix J can be decomposed into the summation of two symmetric unitary matrix if the singular values of J are all less than or equal to 2.

Proof: With singular value decomposition, one can factorize any arbitrary 2×2 matrices J in the form

$$J = W\Sigma V^{\dagger} \qquad \text{S3.3}$$

where W and V are both unitary matrix, and Σ is a real diagonal matrix, $\Sigma = \begin{bmatrix} r_1 & 0 \\ 0 & r_2 \end{bmatrix}$, r_1 and r_2 are nonnegative real numbers and called as the singular values of J.

The columns of W (left-singular vectors) and V (right-singular vectors) are the eigenvectors of $JJ^{\dagger}$ and $J^{\dagger}J$, respectively. Assume that $J^{\dagger}J\mathbf{x}_1 = \lambda\mathbf{x}_1$ and $JJ^{\dagger}\mathbf{x}_2 = \lambda\mathbf{x}_2$. If J is symmetric, we have $J^{\dagger} = \bar{J}^T = \bar{J}$, where J^T denotes the matrix transpose of J and $\bar{J}$ is the conjugate of J, then $J^{\dagger}J\mathbf{x}_1 = \bar{J}J\mathbf{x}_1 = \lambda\mathbf{x}_1$ and

$JJ^\dagger \mathbf{x}_2 = J\bar{J}\mathbf{x}_2 = \lambda \mathbf{x}_2$. So $\bar{J}J\bar{\mathbf{x}}_2 = \lambda \bar{\mathbf{x}}_2$. Therefore, we can choose $\mathbf{x}_1 = \bar{\mathbf{x}}_2$, then $W = \bar{V}$. Indeed, for symmetric matrices, Takagi decomposition is a special case of the singular value decomposition. Takagi tell us there is a unitary W such that $J = W\Sigma W^T$ if J is a symmetric matrix, Again, we have $V^\dagger = W^T$, i.e., $\bar{V} = W$.

It can be proved that any complex number r can be decomposed as two complex numbers with unit amplitude when $|r| \le 2$. Therefore, if the singular values of J are all less or equal to 2, i.e., $r_1, r_2 \le 2$, we have

$$J = W\Sigma V^\dagger = W\Sigma W^T = W\begin{bmatrix} e^{i\varphi_1} + e^{i\varphi_3} & 0 \\ 0 & e^{i\varphi_2} + e^{i\varphi_4} \end{bmatrix} W^T$$

$$= W\begin{bmatrix} e^{i\varphi_1} & 0 \\ 0 & e^{i\varphi_2} \end{bmatrix} W^T + W\begin{bmatrix} e^{i\varphi_3} & 0 \\ 0 & e^{i\varphi_4} \end{bmatrix} W^T \qquad \text{S3.4}$$

As W is unitary, the two terms $W\begin{bmatrix} e^{i\varphi_1} & 0 \\ 0 & e^{i\varphi_2} \end{bmatrix} W^T$ and $W\begin{bmatrix} e^{i\varphi_3} & 0 \\ 0 & e^{i\varphi_4} \end{bmatrix} W^T$ are both symmetric unitary matrixes, too. According to Theorem 1, they must can be factorized in the form $R(-\theta_1)\begin{bmatrix} e^{i\varphi_1} & 0 \\ 0 & e^{i\varphi_2} \end{bmatrix} R(\theta_1)$.

According to Theorems 1 and 2, the precondition for the validity of Eq. S3.1 (i.e., Eq. 2 in the main text) is that the singular values of the symmetric Jones matrix J are both no more than 2. The singular values (r_1 and r_2) of J are the square roots of eigenvalues (λ) from $J^\dagger J$, which can be solved by

$$\det\left(J^\dagger J\text{-}\lambda I\right) = \lambda^2 - \left(|a|^2 + 2|b|^2 + |c|^2\right)\lambda + \left|ac - b^2\right|^2 = 0 \qquad \text{S3.5}$$

To ensure $r_1, r_2 \le 2$, both the two eigenvalues $\lambda_1, \lambda_2 \le 4$. Then we have

$$\begin{cases} 4^2 - 4\left(|a|^2 + 2|b|^2 + |c|^2\right) + \left|ac - b^2\right|^2 \geq 0 & \text{S3.6.1} \\ \left(|a|^2 + 2|b|^2 + |c|^2\right)/2 \leq 4 & \text{S3.6.2} \end{cases}$$

We define $f(a,b,c) = 4^2 - 4\left(|a|^2 + 2|b|^2 + |c|^2\right) + \left|ac - b^2\right|^2$, and its minimum value is

$$\begin{aligned} f(a,b,c)_{\min} &= 4^2 - 4\left(|a|^2 + 2|b|^2 + |c|^2\right) + \left(|a||c| - |b|^2\right)^2 \\ &= \left(|b|^2 - |a||c| - 4\right)^2 - 4\left(|a| + |c|\right)^2 \\ &= \left(|b|^2 - \left(|a| - 2\right)\left(|c| - 2\right)\right)\left(|b|^2 - \left(|a| + 2\right)\left(|c| + 2\right)\right) \geq 0 \end{aligned} \quad \text{S3.7}$$

According to Eq. S3.6.2, $\left(|b|^2 - \left(|a| + 2\right)\left(|c| + 2\right)\right) \leq 0$, then we have

$$\begin{cases} \left(|b|^2 - \left(|a| - 2\right)\left(|c| - 2\right)\right) \leq 0 & \text{S3.8.1} \\ \left(|a|^2 + 2|b|^2 + |c|^2\right)/2 \leq 4 & \text{S3.8.2} \end{cases}$$

One simple sufficient condition for the above two inequations is

$$\begin{cases} |a| + |b| \leq 2 \\ |b| + |c| \leq 2 \end{cases} \quad \text{S3.9}$$

Note that the Jones matrix Eq. S3.1 is not normalized for simplicity of expression. Therefore, the singular values of Jones matrix must be no more than 2, corresponding to a unity transmission. For our designed two layer metasurfaces, their Jones matrix components are confined by defining the following boundary loss:

$$\begin{aligned} L_{bnd} &= \mathrm{Re}LU\left(\left|J_{11}^1\right| + \left|J_{12}^1\right| - 2\right) + \mathrm{Re}LU\left(\left|J_{22}^1\right| + \left|J_{12}^1\right| - 2\right) \\ &\quad + \mathrm{Re}LU\left(\left|J_{11}^2\right| + \left|J_{12}^2\right| - 2\right) + \mathrm{Re}LU\left(\left|J_{22}^2\right| + \left|J_{12}^2\right| - 2\right) \end{aligned} \quad \text{S3.10}$$

where $ReLU$ is the rectified linear unit. For numerical calculation, the metasurface layer is divided into pixels and the loss is then averaged over all pixels. The gradients of this boundary loss with respect to the Jones matrix components of the two single layers can be readily obtained.

S4. Design of the target equivalent Jones matrix and comparison with optimized results

The designed target equivalent Jones matrix has four components with each having two degrees of freedoms (DOFs), amplitude and phase. Four nanoprinting images (weather symbol images, three intensity levels) and four holographic images (letter strings "XX", "XY", "YX" and "YY") are encoded into the four components of the equivalent Jones matrix to serve as the targets. The four channels are independent, and for each of them, we use a modified Gerchberg-Saxton algorithm to obtain its amplitude and phase distributions as the same as our previous work[4]. The amplitude distribution of the equivalent Jones matrix is chosen as that of the input nanoprinting image. Then a random phase distribution is added to it and propagated to the holographic image plane using Rayleigh–Sommerfeld diffraction method that propagates in silicon dioxide substrate with refractive index of 1.45. The amplitude of the holographic image is then substituted with the designed one, and propagated backward to the nanoprinting plane. At the nanoprinting plane, the amplitude constraint is also taken. After several iterations, when the computed holographic amplitude at the far-field holographic plane is close to the target ones, we can obtain the phase distribution of the equivalent Jones matrix.

The operating wavelength is designed at 808 nm and the sampling period is chosen as 800 nm to avoid high order diffractions. The design nanoprintings have 320×320 pixels with a total size of $256\mu m \times 256\mu m$. The holographic images are designed at

2500 μm above the nanoprinting in glass substrate, corresponding to a maximal numerical aperture about $n\sin\theta \approx 1.45\times 0.1 = 0.145$. Usually, a smaller numerical aperture indicates a slower fluctuation of the phase distribution, i.e., smaller phase difference between adjacent pixels. We choose such a long nanoprinting-holography distance to ensure that the small phase fluctuation do not strongly affect the observation of nanoprinting images.

Considering the convenience in the experimental measurement, we set the vertical distance between the two single layer metasurfaces as 150 μm. The material between the two layers is air with refractive index 1.0. The targets of the amplitude and phase distributions of the four components of the equivalent Jones matrix are shown in Figure S2, where the optimized results based on the gradient descent optimization method are also shown. It can be observed that the phase distributions between the target and optimized results are almost identical, while the amplitude distributions differ a little bit around the boundaries. The reason is that the amplitude at the boundary has a sharp fluctuation including high angular spectrum frequencies which is beyond our design (Note that our design has a numerical aperture of 0.145). From this aspect, we can see that the optimized results agree very well with the target ones, demonstrating the feasibility of the gradient descent optimization to obtain the arbitrary equivalent Jones matrix.

When a complex field is imaged, the phase distribution can cause a highly speckly image due to the destructive interference of adjacent pixels. Therefore, the imaged

amplitude fields may differ from the calculated amplitude distributions. The bottom row in Figure S2 shows the simulated amplitude distributions with N.A.=0.25, where some artifacts can be observed due to the phase singularities that generally vary across 2π around a contour circling.

The optimal input values, i.e., the Jones matrixes of the two single layers are provided in Figure S3. The amplitudes of all components do not exceed over 2 due to the introducing of the boundary constraint loss.

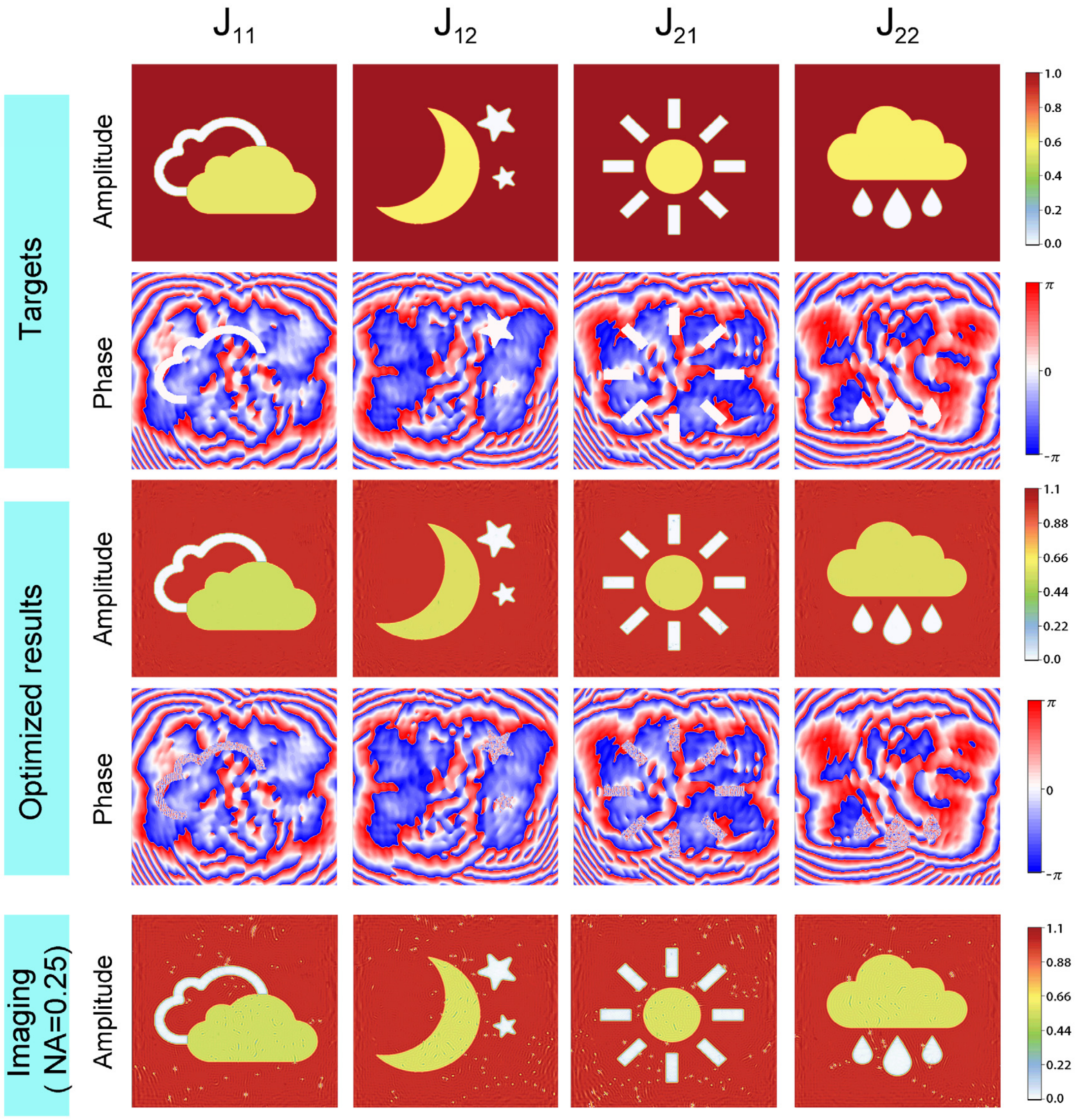

Figure S2. The designed targets, the optimized results and the imaged images (N.A.=0.25) of

the four components of the equivalent Jones matrix.

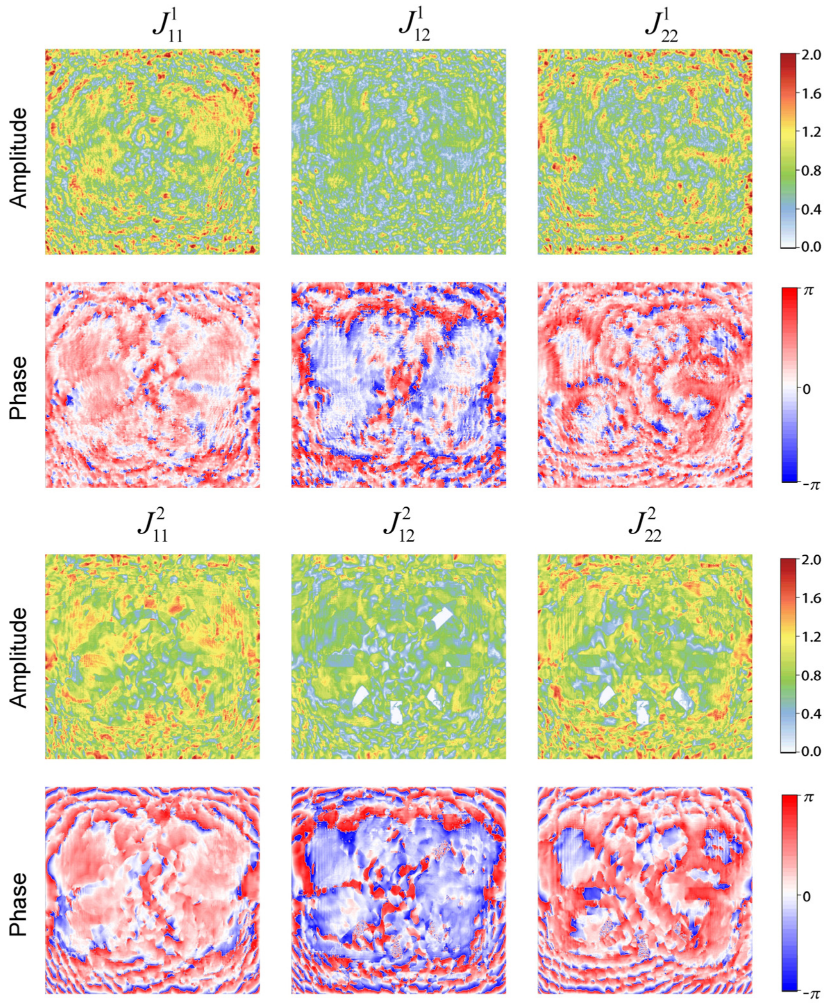

Figure S3. Optimized input variables of the Jones matrix of the two single layers from the gradient descent algorithm. The non-diagonal entries of the Jones matrix are the same due to the symmetry of single layer and therefore we only present the 12th component here.

S5. Metasurface unit design

As outlined in the main text, the symmetric Jones matrix of the single layer with six DOFs can be constructed by two nano pillars in one unit. The nano pillars are made of crystal-silicon with a fixed height of 600 nm on glass substrate. A schematic view of

such nano pillar is shown in Figure S4 (a). Due to the symmetry, the unit cell support two propagation modes along its *x* and *y* axis. The transmission magnitude and phase shift introduced by the nano pillar with *x*-polarized and *y* polarized incidences, as a function of the transverse dimensions of the nano pillars d*x* and d*y*, are shown in Figure S4 (b-e). The results are obtained numerically via FDTD simulations. Note that the two layer metasurfaces are arranged front-to-front to maintain a homogeneous air environment between them. The transmission properties of nanopillars of the two layers (one is from substrate to air and the other is opposite) are the same due to reciprocity principle and can be extracted from the same database.

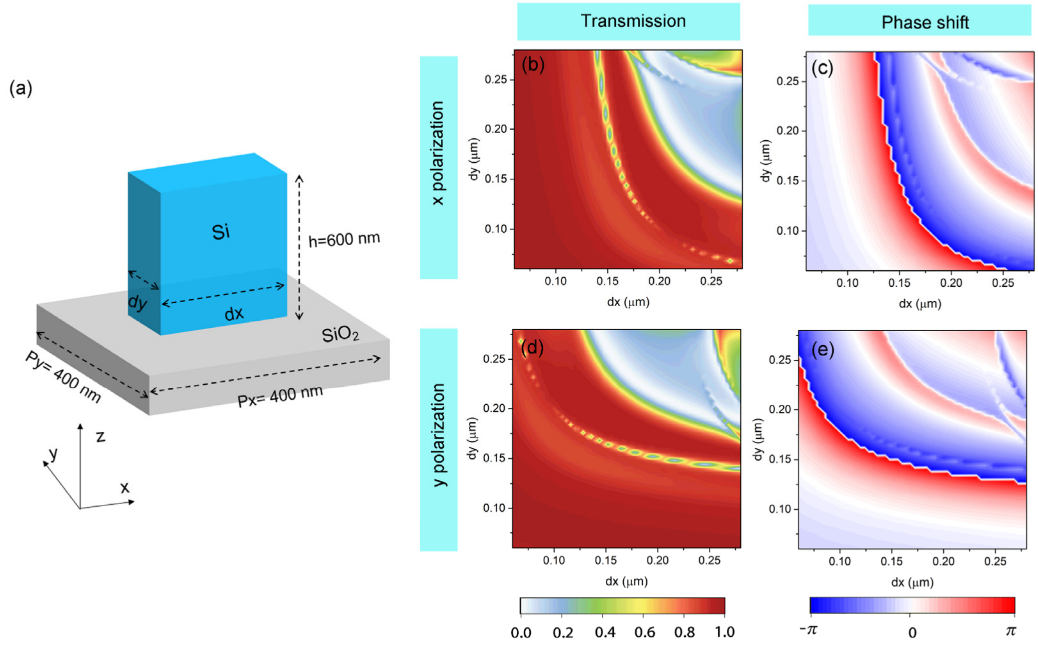

Figure S4. Metasurface unit design. (a) Schematic of the metasurface unit cell composed of a rectangle nanopillar made of silicon (Si) standing on a glass substrate. The period along *x* and *y* direction is 400 nm, and the height is fixed of 600 nm. (b-c) Normalized transmission (b) and phase response (b, in radians) of the nanopillar at incident wavelength 808 nm as a function of

the transverse dimensions, dx and dy, for incident x-polarization. (d-e) Normalized transmission (d) and phase response (e, in radians) of the nanopillar at incident wavelength 808 nm as a function of the transverse dimensions, dx and dy, for incident y-polarization. The optical response for y-polarization response is obtained by swapping x and y of x-polarization response due to the nature of symmetry.

For a given Jones matrix with six DOFs, we can extract the rotational angles and the phase shifts along *x* and *y* directions of both nano pillars, according to Eq. S3.1. For each individual nano pillar, the transverse dimensions are chosen as follows[5]: (1) first set a pre-defined average transmission magnitude t_{avg} . This value is basically determined by the overall transmissions of the nano pillar (Figure S4b) with different transverse dimensions and should not be small. In our work, we choose $t_{avg} = 0.98$. (2) calculate the complex-valued errors $\varepsilon_x = \left| t_{avg} e^{i\varphi_{x,desired}} - t_{simulated} e^{i\varphi_{x,simulated}} \right|$ and $\varepsilon_x = \left| t_{avg} e^{i\varphi_{x,desired}} - t_{simulated} e^{i\varphi_{x,simulated}} \right|$, and choose the large one $\varepsilon_{\max} = \max(\varepsilon_x, \varepsilon_y)$. (c) determine the configuration that minimizes $\varepsilon_{\max}$ for all possible dimensions.

The results in Figure S4 are simulated at normal incidence. We choose several nanopillars with different dimensions and perform their optical responses under different incident angles. The results in Figure S5 show that the optical response remains almost unchanged with the incident angle, therefore can be used for cases with oblique incidences.

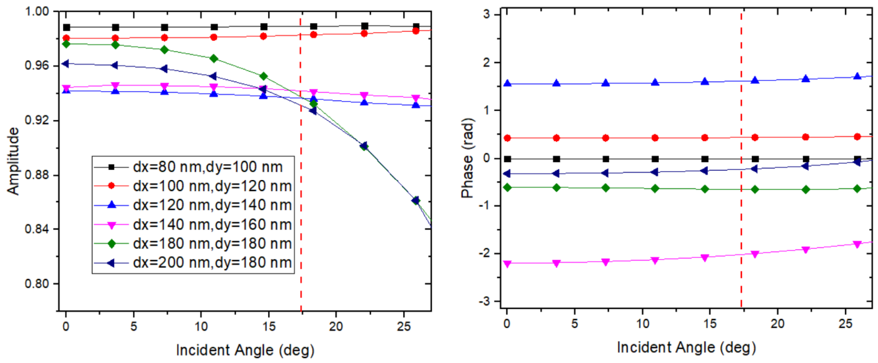

Figure S5. Amplitude and phase shift of nanopillars with different dimensions as a function as incident angles. The red dashed line indicates the position of incident angle of $k_x = 0.3k$. The optical response of the nanopillar remains almost unchanged with the incident angle.

S6. FDTD simulation of the realistic structures with different optical setups

The numerical calculations based on the diffraction theory in Figure S2 show good results. However, for realistic structures, many factors, such as the errors between the designed phase shifts and real ones of the nano pillars, the residual zero order diffractions and the coupling between nano pillars may all affect the optical performances. Therefore, it is necessary to perform full wave electromagnetic simulations (FDTD simulations) with the realistic structures, which can also provide a guidance for design and measurement. The structure in our work consists of two layer metasurfaces, with a side length of 256 μm and a vertical distance of 150 μm. Obviously, it is unrealistic to simulate the whole structure and thus we turn to a scaled-down version with 120×120 pixels (96 μm $\times$ 96 μm) and gap distance of 100 μm. The holographic images are designed at a shorter distance of 400 μm above the second layer.

It is still impossible to simulate the whole structure as the dimensions along the three directions are all around hundreds of micrometers. As the Jones matrix of each metasurface layer varies spatially and cannot reaching unity transmission, some light energy must be reflected as the period is smaller than the operation wavelength. We simulate the reflections of the two metasurface layers, which are 50% and 20%, respectively. Therefore, the light impinging on the second layer from the twice reflection between the two layers is 10% of that of the transmission of the first layer. For each component of the Jones matrix, this ratio is reduced by half to 5%. The value is relatively small, and it is reasonable to neglect the reflections between the two layers. Then, the simulation of the whole structure can be divided into three steps: (1) perform the simulation with only the first layer and obtain the near fields at the output plane of the first layer; (2) The equivalent electric and magnetic currents obtained from above near fields are used to calculate the far fields at the incident plane of the second layer, which are then imported as source to excite the second layer. (3) perform the simulation of the second layer with the imported source and obtain the near fields at the output plane of the second layer. With the above treatment, we decompose the simulation of the whole structure into two simulations with a shorter size along the z direction. Each simulation can be performed within an acceptable time (about 10 hours on a workstation with CPU AMD 3990x and RAM 256G).

The obtained near fields at the output plane of the second layer are used to extract the nanoprinting images and holographic images with different numerical aperture

centers and sizes. Assuming that the far fields in the angular spectrum domain are $A(u,v,0)$, the images at distance *h* above the second layer with numerical aperture size *NA* at center (u_0,v_0) can be calculated as

$$H(x_2,y_2)=\iint_{u,v} A(u,v,0)\cdot G(u,v,h)\cdot F(u,v)\cdot \exp(i2\pi(ux_2+vy_2))dudv \qquad \text{(S6.1)}$$

where $G(u,v,h)=\exp(i2\pi h(\lambda^{-2}-u^2-v^2)^{1/2})$ and $F(u,v)$ is the filter in Fourier plane, as

$$F(u,v)=\begin{cases}1 & if\ \sqrt{(u-u_0)^2+(v-v_0)^2}\le \dfrac{NA}{\lambda} \\ 0 & else\end{cases} \qquad \text{(S6.2)}$$

The nanoprinting and holographic images can be obtained by set *h*=0 and *h*=400 μm. We implement different optical setups: normal incidence-normal detection, oblique incidence-normal detection, and oblique incidence-oblique detection. The observation numerical aperture is chosen *NA*=0.2 and the center of the oblique detection is set $(u_0,v_0)=(\frac{0.3}{\lambda},0)$. The full simulated results of the three cases are shown in Figure S6.

It can be observed that the nanoprinting images are strongly affected by the different optical setups. This is because the designed nanoprintings and zeros orders are on the same order of magnitude. When applying the normal incidence normal detection setup, both the zero order diffractions from two layers are detected, which results in a very poor image quality of nanoprinting. By applying the oblique incidence normal detection, only the zero order diffraction from the second layer are detected. The nanoprinting for the oblique incidence oblique detection setup has the highest image quality among the

three setups since the zero order diffractions from both layers are filtered.

On the other hand, the holographic images for all cases can be observed clearly and have similar image fidelities. This is because the magnitudes of the holographic images in design are much higher than that of zero orders, which thus do not strongly affect the holographic images. Due to the limit of computing capacity, the input images in the simulation have a small size and a low resolution. We anticipate that the image qualities can be increased for practical structures that have much larger number of pixels.

As mentioned above, when a complex field is imaged, the phase distribution can cause a highly speckly nanoprinting image due to the destructive interference of adjacent pixels. Therefore, we only choose a three intensity level images as the nanoprinting for demonstration. If the phase distribution is set as uniform, grayscale nanoprinting image can be perfectly constructed (Fig. S7). The fringes arise due to the oblique detection.

Similarly, if we set the phase of the holographic images to be uniform, the fidelity of image can be enhanced. In this case, the four Jones matrix now can be directly calculated by a reverse Rayleigh–Sommerfeld transformation of the input holographic images. Fig. S8 shows a comparison between the uniform-phase holographic images and the previously calculated nanoprinting-holography. The much lower speckles and the calculated phase distributions (almost uniform) show good reconstruction of complex amplitude in the diffraction field with our bilayer metasurface.

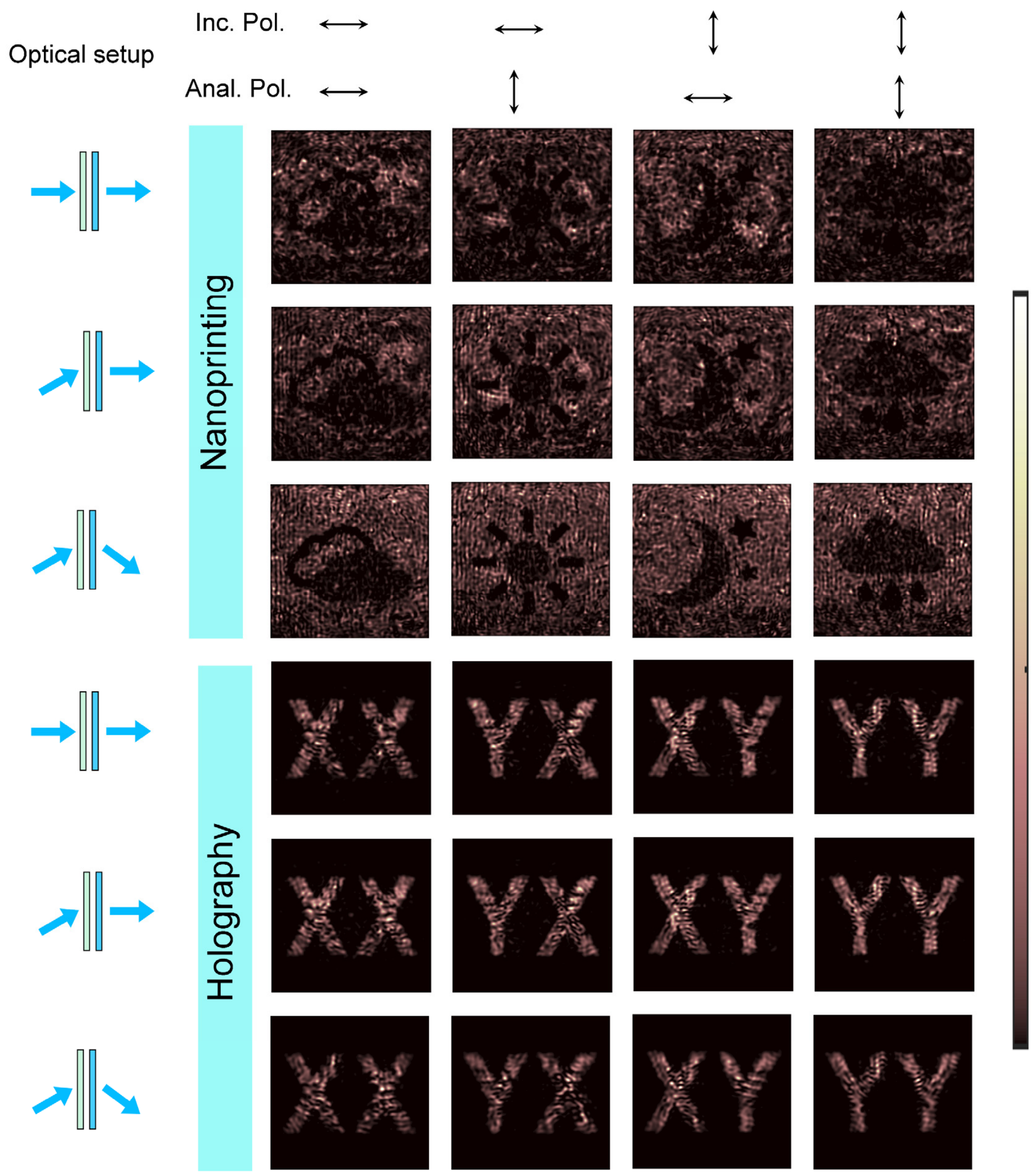

Figure S6. FDTD simulated results of the nanoprintings and holographic images with different optical setups. The schematic views of different optical setups are shown in the left column. The incident and analyzed polarizations are indicated at the top two rows.

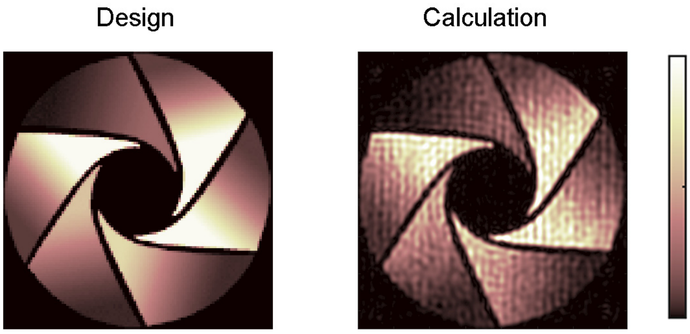

Figure S7. Designed and simulated grayscale nanoprintings. In the design of bilayer metasurface, the phases of the Jones matrix are set as to be uniform.

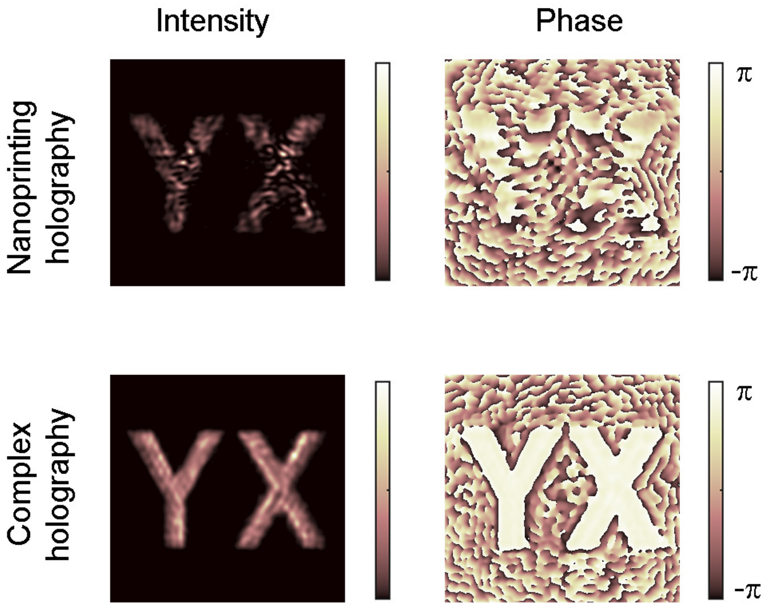

Figure S8. Comparison of the nanoprinting-holography and complex holography. These images are simulated under oblique-incidence oblique-detection strategy. The phase has been subtracted by a term of $k_x x$ (k_x=0.3k_0) induced by oblique detection. In the design of complex holography, the phase is set as uniform, and the simulated phase shows good agreements with design.

S7. Effects of detour phase in multi-element unit design

Figure S9a presents a metasurface unit cell with one element, which imposes amplitude modulation A and phase shift φ on the incident light. If one expects to realize the same optical response by two-element unit design (Figure S9b), a simple way is to use the same two elements as that in Figure S9a. However, this is not always the best choose. For oblique incidence and oblique scattering, the lateral displacements of the two elements introduce detour phases within this unit cell. Assuming that the two elements are evenly distributed that are located at x coordinates of -P/4 and P/4, where P is the period, the summation of the scatterings from the two elements are $Ae^{i\varphi}(e^{i\Delta\varphi}+e^{-i\Delta\varphi})=2A\cos\Delta\varphi e^{i\varphi}$, where $\Delta\varphi=\frac{2\pi}{\lambda}\frac{P}{4}(\sin\theta_1+\sin\theta_2)$, θ_1 is the incident angle and θ_2 is the scattering angle.

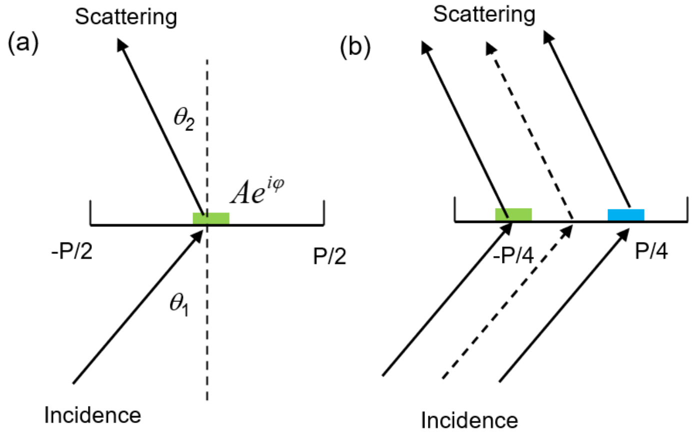

Figure S9. Schematics of the metasurface unit cell with one element and two elements.

It is shown that the duplication of the element in the unit cell do not alter the phase

shift (must be evenly distributed) but impose an amplitude modulation of $2A\cos\Delta\varphi$. To satisfy the Shannon–Nyquist sampling theorem, the scattering angle is limited by $\sin\theta_2 \le \lambda / 2P$. At the limit scattering angle, $\Delta\varphi = \pi / 4$ and the efficiency decreases to $\cos^2\Delta\varphi = 50\%$. It is also obvious that the efficiency will decrease for oblique incidence. If we consider the detour phase and individually design the two elements, the total scattering becomes $Ae^{i(\varphi_1+\Delta\varphi)} + Ae^{i(\varphi_2-\Delta\varphi)}$, where φ_1 and φ_2 are the phase shifts of the two elements. The best optical performance occurs when φ_1=φ-$\Delta\varphi$ and φ_2=$\varphi + \Delta\varphi$, and the total optical field is $2Ae^{i\varphi}$.

As for the unit design in Figure 2c of main text, the above two elements correspond to the nano pillars *AB* and *A*'*B*'. For each pixel of the single layer, a special amplitude modulation and phase shift is designed for each component of its Jones matrix. In our work, the Jones matrix of the nano pillars *AB* and *A*'*B*' are individually designed for oblique incidence (metasurface 1, Figure 2d) or oblique scattering (metasurface 2, Figure 2d) according to the above analysis. A comparison of the FDTD simulations between considering the detour phase (individually designing nano pillars *AB* and *A*'*B*') and without (*A*'*B*' is a duplication of *AB*) is shown in Figure S10. Clearly, the nanoprinting images with the consideration of the detour phase have much higher fidelities than that without the consideration of the detour phase.

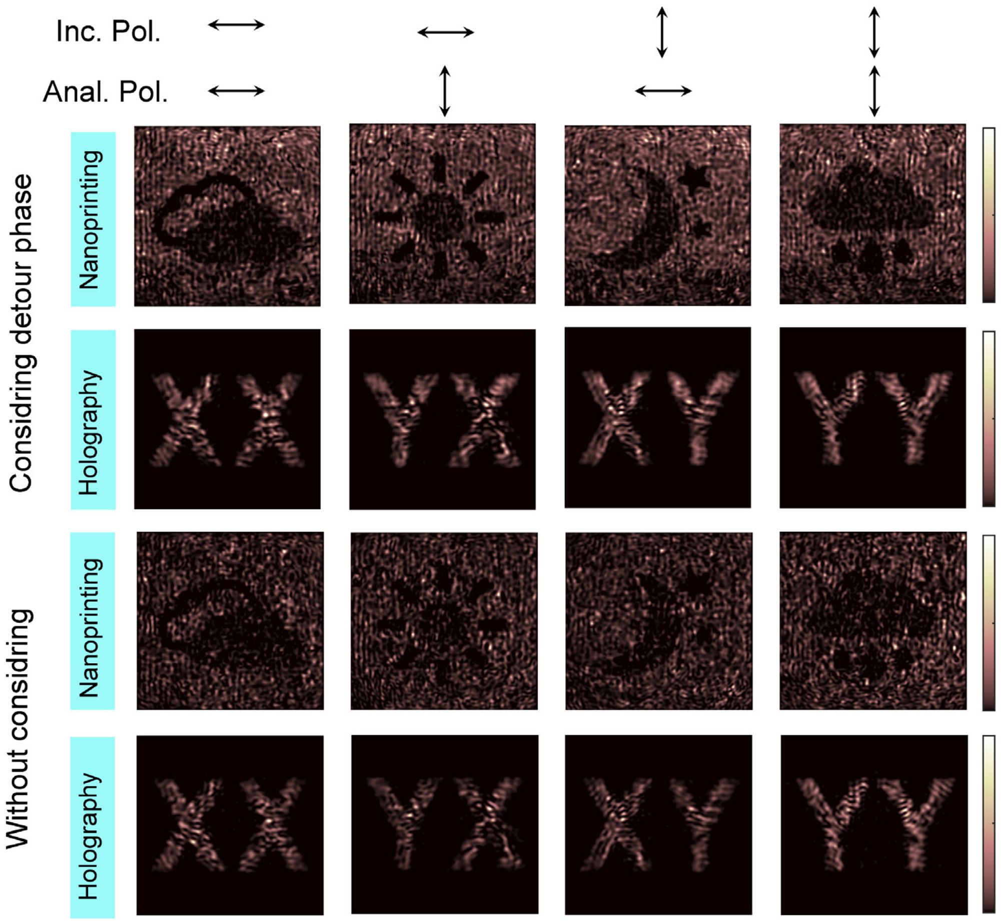

Figure S10. FDTD simulations of the nanoprinting and holographic images with the consideration of the detour phase in the unit pixel (first and second rows) and without (third and fourth rows).

S8. Alignment sensitivity of the two layer metasurfaces

The optical performance of nanoprinting and holographic images are dependent on the alignment of the two layer metasurfaces. Figure S11 shows the simulated results of the images with different translational in-plane x-shift values. Basically, the images can be distinctly observed within a shift value of 5 μm. We define a figure-of-merit, the mean square error (MSE) to characterize the difference between the simulated images

and the targets. The simulated image is multiplied by a varying constant factor to minimize the MSE value. The minimal MSE values under different alignment shifts are shown in Figure S12. One can observe that the MSE reaches to minimum when there is no shift. Due to the non-uniform phase distribution (destructive interference of adjacent pixels) and oblique observation, the nanoprintings present speckles and fringes, making the MSE value relatively high. The MSE of the holography is smaller than nanoprinting images, demonstrating the fidelity of holography is higher than that of nanoprinting. In experiment, the two metasurfaces are mounted on two 3D translational stages (Thorlabs, MBT616D/M), which can provide a resolution about 100~200 nm, thus satisfying our alignment requirement.

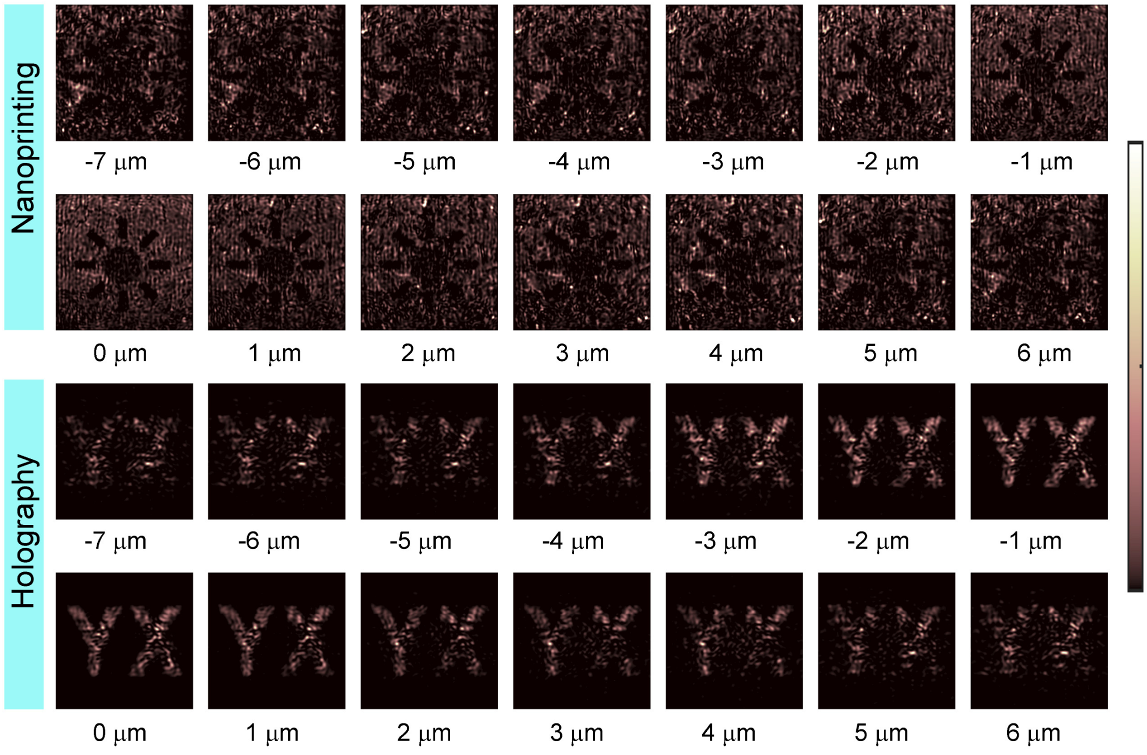

Figure S11. Simulated nanoprinting and holographic images with x-polarized incidence

and y-polarized analyzation for different translational shift values.

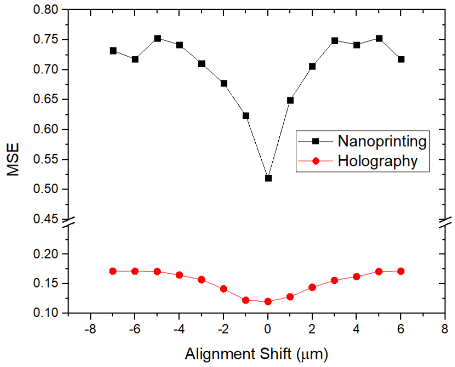

Figure S12. Calculated mean square error (MSE) between the reconstruction images and the targets with different alignment shifts.

S9. Optical setup for measurement

A schematic view of our optical setup with detailed parameters is shown in Figure S13. The focal lengths of the objective (RMS20X-PF, 20X, N.A.=0.5, Thorlabs Inc.), Lens 1, Lens 2 and Lens 3 are f_o=9 mm, f_1=100 mm, f_2=200 mm and f_3=200 mm, respectively. A beam with diffraction angle θ firstly focuses at position of $d=f_o\sin\theta$ away from the center axis at the back Fourier plane of the objective. After passing through Lens 1 and Lens 2, it focuses at the back focal plane of Lens 2 with a distance of $D=f_2/f_1*d=f_2 f_o \sin\theta/f_1$ away from the center axis.

For optical measurement, we apply a phase shift gradient of k_x/k=0.3 on the second layer, corresponding to $\sin\theta$=0.3. Therefore, the center of the filter is located at D=5.4 mm away from the center axis. The aperture size a of the filter is chosen to have a

numerical aperture of N. A.= 0.2, corresponding to a diameter of a=2 $f_2 f_0$ N.A. / f_1 =7.2 mm. In measurement, a continuously variable iris is used for the filter.

To measure the efficiency, we place a power meter (S120C, Thorlabs, Inc.) behind the filter, so that only the light contributing to the holography can be detected. The incident laser is collimated with uniform intensity in the center with a beam expander to measure the energy density per area. The incident power is then calculated as the product of the energy density per area and the metasurface areas. The efficiency is defined as the ratio between the measured holographic power and the incident power. Additionally, the effects of the light absorptions through the substrate, objective and the lenses in the optical setup are taken into consideration in the final calculated efficiency.

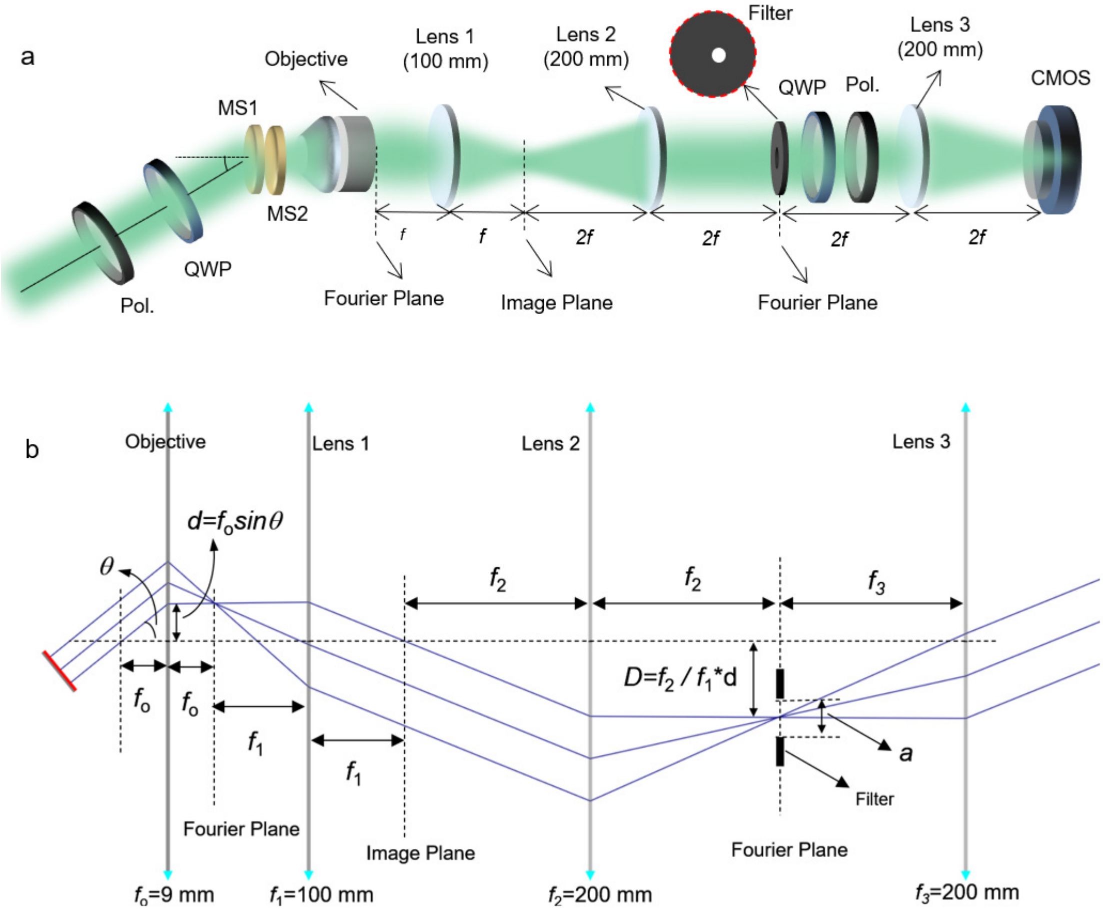

Figure S13. a, Schematic of the optical setup for measurements; b, Schematic of the propagation of a plane wave at oblique incidence, showing the detailed parameters for spatial filtering.

S10. Calculation of optical responses with rotation between the two layer metasurfaces

Here, we investigate the optical responses of the two layer system with different rotation angle. The first layer is assumed to be fixed, and the second layer is rotated anticlockwise with an angle ϕ. The Jones matrix components of the second layer after rotation are given by:

$$\begin{bmatrix} J_{11}^{2\phi}(x_2,y_2), J_{12}^{2\phi}(x_2,y_2) \\ J_{21}^{2\phi}(x_2,y_2), J_{22}^{2\phi}(x_2,y_2) \end{bmatrix} = \begin{bmatrix} J_{11}^{2}(x_2,y_2), J_{12}^{2}(x_2,y_2) \\ J_{21}^{2}(x_2,y_2), J_{22}^{2}(x_2,y_2) \end{bmatrix} \quad \text{(S10.1)}$$

for $\phi = 0°$,

$$\begin{bmatrix} J_{11}^{2\phi}(x_2,y_2), J_{12}^{2\phi}(x_2,y_2) \\ J_{21}^{2\phi}(x_2,y_2), J_{22}^{2\phi}(x_2,y_2) \end{bmatrix} = \begin{bmatrix} J_{22}^{2}(-y_2,x_2), -J_{21}^{2}(-y_2,x_2) \\ -J_{12}^{2}(-y_2,x_2), J_{11}^{2}(-y_2,x_2) \end{bmatrix} \quad \text{(S10.2)}$$

for $\phi = 90°$,

$$\begin{bmatrix} J_{11}^{2\phi}(x_2,y_2), J_{12}^{2\phi}(x_2,y_2) \\ J_{21}^{2\phi}(x_2,y_2), J_{22}^{2\phi}(x_2,y_2) \end{bmatrix} = \begin{bmatrix} J_{11}^{2}(-x_2,-y_2), J_{12}^{2}(-x_2,-y_2) \\ J_{21}^{2}(-x_2,-y_2), J_{22}^{2}(-x_2,-y_2) \end{bmatrix} \quad \text{(S10.3)}$$

for $\phi = 180°$,

$$\begin{bmatrix} J_{11}^{2\phi}(x_2,y_2), J_{12}^{2\phi}(x_2,y_2) \\ J_{21}^{2\phi}(x_2,y_2), J_{22}^{2\phi}(x_2,y_2) \end{bmatrix} = \begin{bmatrix} J_{22}^{2}(y_2,-x_2), -J_{21}^{2}(y_2,-x_2) \\ -J_{12}^{2}(y_2,-x_2), J_{11}^{2}(y_2,-x_2) \end{bmatrix} \quad \text{(S10.4)}$$

for $\phi = 270°$, where the superscript ϕ indicates the rotation case, and the Jones matrix components at the right hand side of the above formulas are the ones without rotation, i.e., the initial input variables in the gradient descent optimization algorithm. Note that both the Jones matrix component values and the coordinates are changed after rotation. According to Eq. S1.5, the equivalent Jones matrix after rotation is given by

$$J_{mn}^{\phi}(x_2,y_2) = \sum_{q=1,2} J_{mq}^{2\phi}(x_2,y_2) \iint_{x_1,y_1} J_{qn}^{1}(x_1,y_1) \cdot f(x_2-x_1, y_2-y_1, z) dx_1 dy_1 \quad \text{(S10.5)}$$

The holographic image with a distance h above the second layer is

$$H_{mn\phi}(x_3,y_3) = \iint_{x_2,y_2} J_{mn}^{\phi}(x_2,y_2) \cdot f(x_3-x_2, y_3-y_2, h) dx_2 dy_2 \quad \text{(S10.6)}$$

where m, n=1 indicates the x-polarized incidence or analyzation, m, n=2 indicates y-polarized incidence or analyzation. Therefore, there are 16 cases by combining the four rotation angles and the four incidence-analyzation polarizations.

For numerical calculation, all the planes are sampled to $N \times N$ equidistant grids with sampling intervals of $\Delta x = \Delta y = P$. We define a loss of

$$L = \frac{1}{16N^2} \sum_{\phi=1}^{4} \sum_{m=1}^{2} \sum_{n=1}^{2} \sum_{i=1}^{N} \sum_{j=1}^{N} \left(\left| H_{mn\phi}(x_{3i}, y_{3j}) \right| - \left| H^t_{mn\phi}(x_{3i}, y_{3j}) \right| \right)^2 \qquad \text{(S10.7)}$$

where we use the index $\phi = 1, 2, 3, 4$ to represent the cases with rotational angle of ϕ= 0°,90°, 180°, 270°, $H^t_{mn\phi}$ indicates the designed target holographic images under *mn* incidence-analyzation polarizations and rotational angle ϕ. Such definition of the loss does not impose any constraints on the phases of the predicted holographic images. The boundary constraint loss is also added in the total loss. The gradient calculations of this loss are more complicated than that of the EQJM, but the basic ideas are the same, the details of which are not present here anymore. Note that there is a freedom of the magnitude ratio for the target images, and we set the maximal amplitude value as 2 for the balance consideration of the astringency and the efficiency.

We input 16 holographic images as the targets, and the optimized results of the gradient descent optimization are shown in Figure S14, which agrees well with our targets.

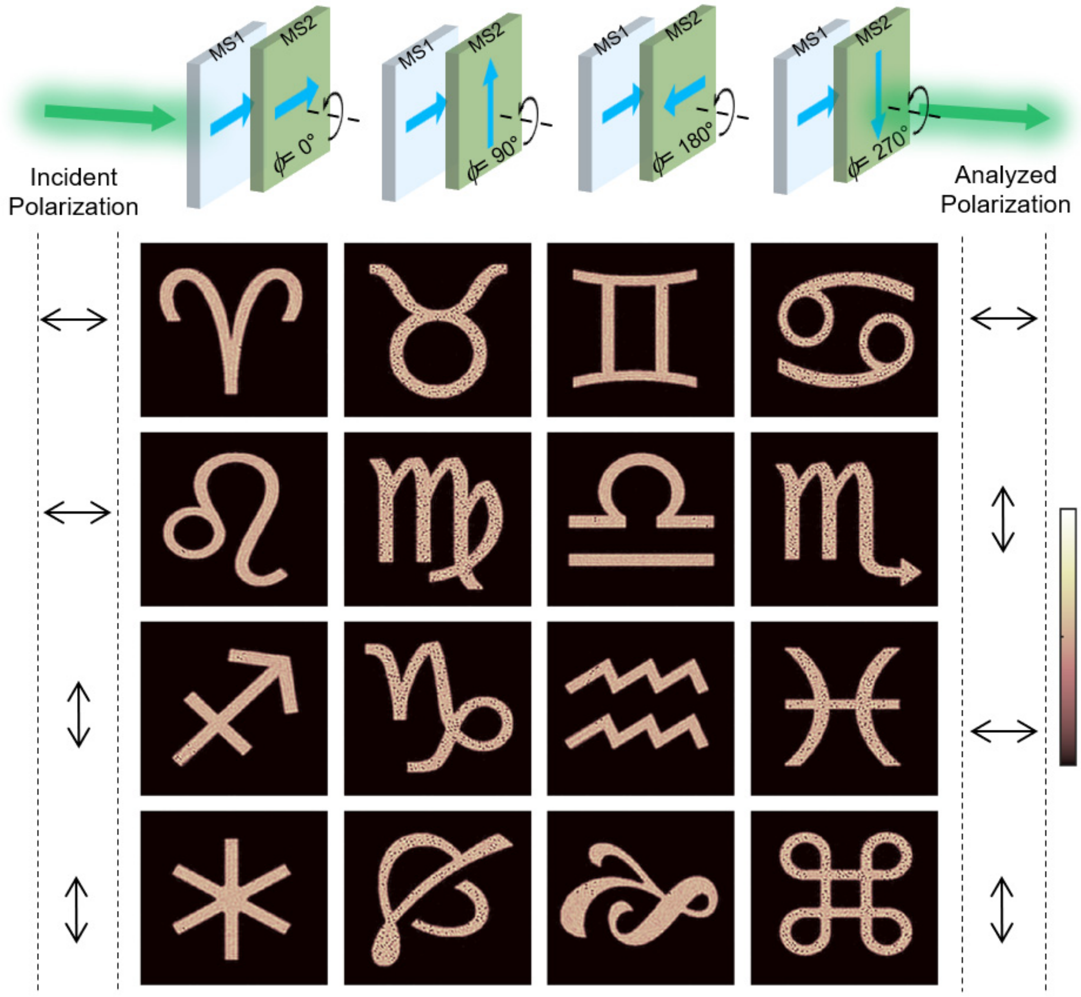

Figure S14. Optimized holographic images with the combinations of different rotation angles, incident polarizations and analyzed polarizations. The incident and analyzed polarizations are indicated at left side and right side, respectively.

S11. Optical performance of the two layer metasurfaces designed with different gap distance

To show the performance of our design strategy with smaller gap distance, we choose several cases with smaller gap distance of z=5 μm, 10 μm , 20 μm and 100 μm, and the corresponding Jones matrixes of each single layer are calculated with gradient descent

algorithm. The results are then simulated by FDTD, with the same procedures as that in supplementary section 6. Figure S15 shows the simulated nanoprintings and holographic images of the four cases. The qualities of holographic images are almost not influenced by the gap distance. Note that the design principle of the bilayer metasurface is based on the far field situation, e.g., the calculation of transmission and phase of single nanopillar (zero order), the Rayleigh–Sommerfeld diffraction between two layer metasurfaces. When the distance between the two layers is decreased to 10 microns or less, it is not far enough to be considered as far field, but the intermediate field. In this situation, the design principle may not be accuracy and introduce errors. Therefore, the fidelities of nanoprintings decrease as the gap distance reduces. Despite of that, for the smallest distance of 5 μm, the simulated results still show distinct nanoprintings as the designed ones, demonstrating the generality of our design strategy.

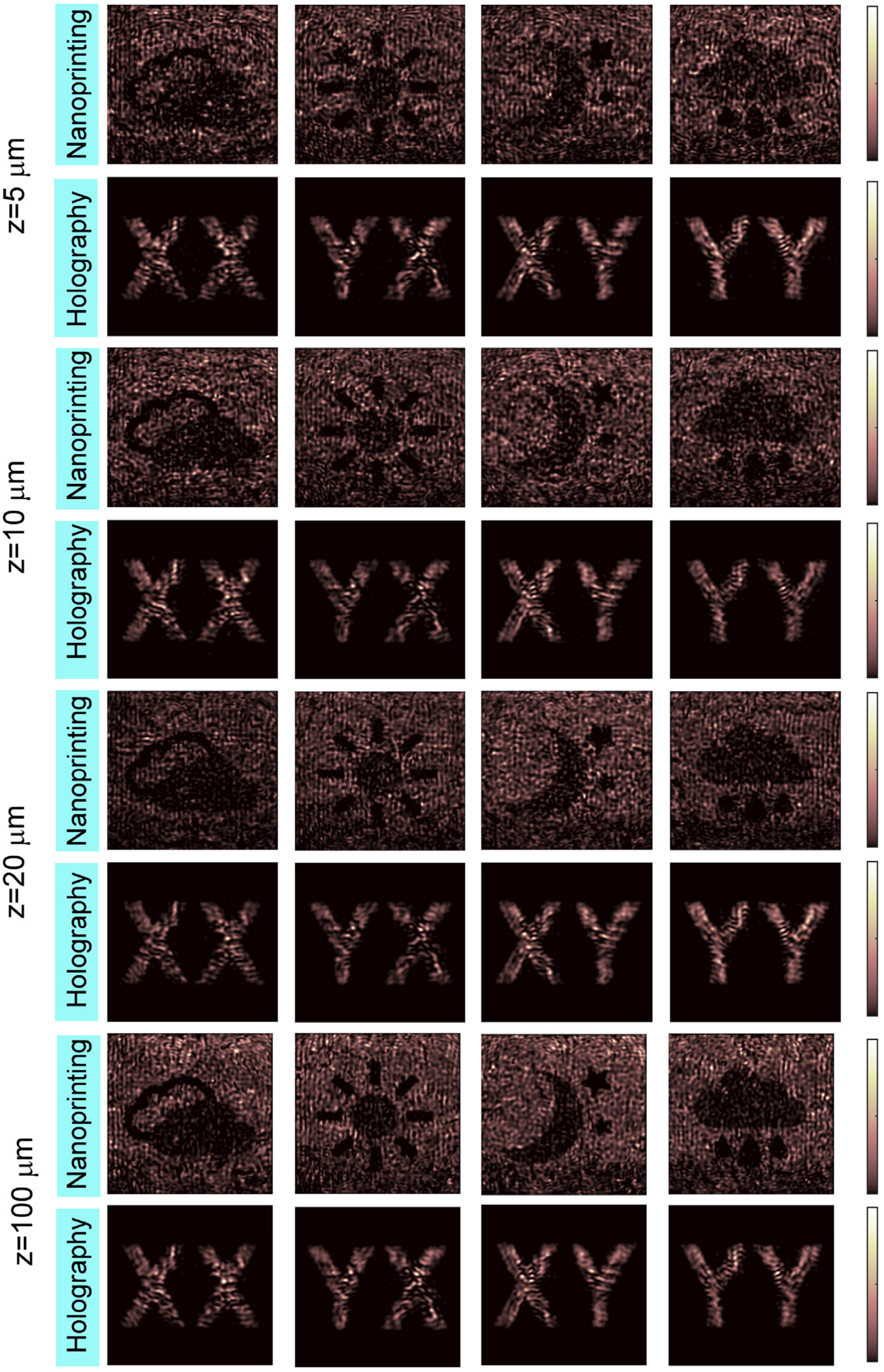

Figure S15. Simulated results of the nanoprinting and holographic images designed with different gap distances. The other geometric parameters are the same as that in supplementary section 6.

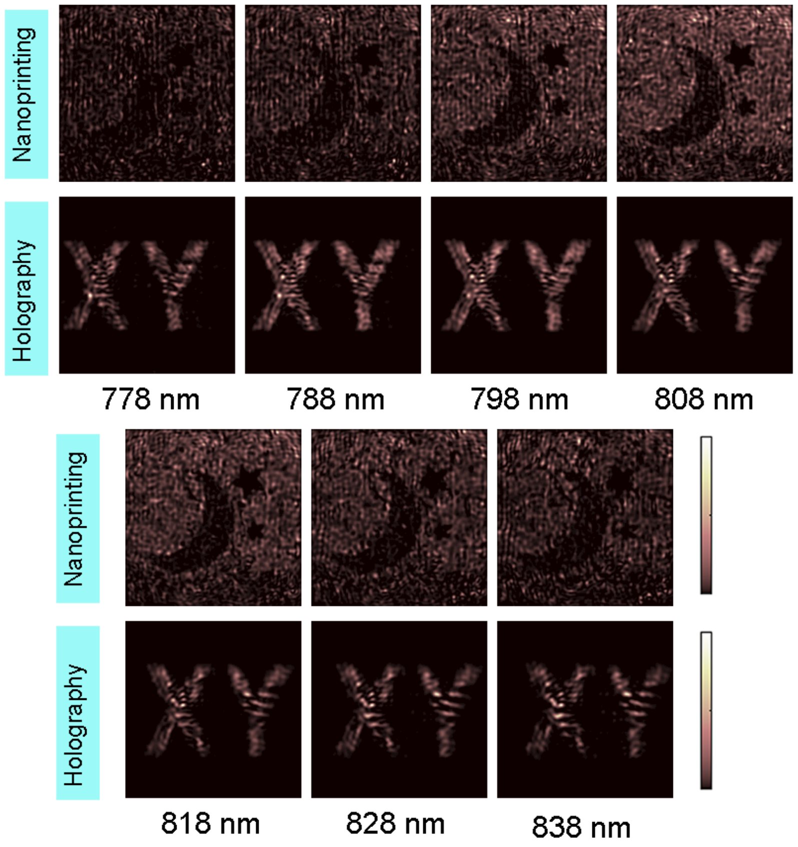

Figure S16. Simulated nanoprinting and holographic images with *y*-polarized incidence and x-polarized analyzation for different incident wavelengths. The bilayer metasurface is designed at 808 nm.

References:

[1] F. B. Shen, A. B. Wang, *Appl. Opt.* **2006**, *45*, 1102.

[2] K. Matsushima, T. Shimobaba, *Opt. Express* **2009**, *17*, 19662.

[3] A. H. Dorrah, N. A. Rubin, A. Zaidi, M. Tamagnone, F. Capasso, *Nat. Photon.* **2021**,

15, 287.

[4] Y. Bao, Y. Yu, H. Xu, C. Guo, J. Li, S. Sun, Z.-K. Zhou, C.-W. Qiu, X.-H. Wang, *Light Sci. Appl.* **2019**, *8*, 95.

[5] J. P. Balthasar Mueller, N. A. Rubin, R. C. Devlin, B. Groever, F. Capasso, *Phys. Rev. Lett.* **2017**, *118*, 113901.
